# Response to diazepam in children with malaria induced seizures

**DOI:** 10.1016/j.eplepsyres.2008.08.002

**Published:** 2008-12

**Authors:** M.L. Ikumi, S.N. Muchohi, E.O. Ohuma, G.O. Kokwaro, C.R.J.C. Newton

**Affiliations:** a Kenya Medical Research Institute (KEMRI)/Welcome Trust Research Programme, Centre for Geographic Medicine Research (Coast), Kilifi, Kenya; b Department of Pharmaceutics and Pharmacy Practice, School of Pharmacy, College of Health Sciences, University of Nairobi, Nairobi, Kenya; c Neurosciences Unit, Institute of Child Health, University College London, United Kingdom; d London School of Hygiene and Tropical Medicine, London, UK

**Keywords:** Anticonvulsants, Benzodiazepine receptor, Diazepam, Malaria, Seizures, Children

## Abstract

Malaria infection reduces the binding capacity of benzodiazepine receptors in mice. We studied the efficacy of diazepam terminating seizures in children with falciparum malaria. Diazepam stopped seizures in fewer patients with malaria parasitaemia (*χ*^2^ = 3.93, *P* = 0.047) and those with clinical diagnosis of malaria (*χ*^2^ = 9.84, *P* = 0.002) compared to those without. However malaria was not identified as an independent risk factor for diazepam's failure to stop seizures in children.

## Introduction

Acute seizures are common in many children presenting to sub-Saharan African health facilities (Idro et al., 2008; Ogutu et al., 2002). In these facilities, diazepam is used as the standard antiepileptic drug (AED) to stop acute seizures and status epilepticus in children, due to its rapid action, low expense and wide availability (Mpimbaza et al., 2008; Ogutu et al., 2002). Falciparum malaria is a common cause of acute seizures in children admitted to hospital (Waruiru et al., 1996) and its metabolic and circulatory complications may affect the action of drugs. About a third of patients with malaria and seizures have cerebral malaria (Asindi et al., 1993).

While falciparum malaria does not appear to alter the pharmacokinetics of diazepam (Ogutu et al., 2002), *Plasmodium berghei* infection in mice significantly decreases the binding capacity of Gamma-aminobutyric acid-A (GABA-A) receptors to which benzodiazepines bind (Kokwaro et al., 1997). Similar studies on effect of malaria on benzodiazepine receptors have not been reported in humans.

We assessed the efficacy of intravenous diazepam which is the preferred route of diazepam administration (Ogutu et al., 2002), in treatment of acute seizures in children with falciparum malaria.

## Methods

### Study population

The notes of children presenting to the pediatric ward of Kilifi District Hospital, Kenya with acute convulsions between 1st March 2000 and 28th February 2007 were retrospectively evaluated. During this period the clinical and laboratory features were recorded systematically on standard proformas. These children were from Kilifi district—a malaria endemic area where individuals are exposed to a range of <1–120 infectious bites per year (Mbogo et al., 2003). However the clinical presentation of malaria is dependent on factors such as age level of transmission and immunity of the patient (Mwangi et al., 2005; Reyburn et al., 2005).

Malaria was considered to be the primary diagnosis if the child had fever and peripheral parasitaemia, without any other cause of the fever found. Information retrieved on the children with convulsions included; age, sex, weight, AED, dose, time of AED administration, number and type of seizures, time the seizures started and stopped, history of seizures, clinical diagnosis, conscious level for patients with cerebral malaria as defined by a Blantyre Coma Scale of ≤2 (Molyneux et al., 1989), oxygen saturation and falciparum malaria parasitaemia (detected in blood slides stained with 10% Giemsa).

All children who were given AEDs for acute convulsions were identified. Children with inadequate/missing information or who had not been given any AED (seizures lasted <5 min and aborted spontaneously) were excluded.

Either intravenous (IV) diazepam (0.3 mg/kg) or intramuscular (IM) paraldehyde (0.4 ml/kg) was used as first line treatment, usually dependent upon the availability of venous access. In the event of refractory seizures, a repeat dose of either drug could be given. If the seizure did not stop, then second line IV phenobarbital (15 mg/kg) or IV phenytoin (18 mg/kg) and eventually the third line (thiopental; 4 mg/kg) were given, according to the hospital protocol (Ogutu et al., 2002) based on ILAE guidelines (1993).

Diazepam was considered to be effective if it stopped the convulsion in less than 15 min with no recurrence (Qureshi et al., 2002). The point in the treatment protocol at which the seizure was terminated as well as factors such as respiratory depression (defined as low oxygen saturation <92% following drug administration) and the effect of malaria as the clinical diagnosis, on the efficacy of AEDs were evaluated.

### Statistical analysis

The data were entered and tabulated in *Microsoft Visual Fox-Pro* 6.0 (Microsoft Corporation) and analysed with Stata (version 9; Stata Corp, College Station, TX, USA). Measures of association were done using Pearson's Chi-square test. We performed a univariate analysis of the various risk factors (history of seizures, seizure duration and malaria as an underlying condition) and stratified for possible confounders such as age and sex. A multiple logistic regression was performed adjusting for history of seizures, age, sex, seizure duration and malaria as an underlying condition.

## Results

A total of 1653 children aged 0–12.9 years, admitted during the 7-year period were given AED to stop acute seizures, of whom 1189 (72%) had adequate information recorded in the notes and proforma. Malaria was the most common underlying condition, occurring in 566 (48%) of children of whom 239 (42%) had cerebral malaria.

First line AEDs stopped seizures in 298 (25%) of all patients. Diazepam terminated seizures in fewer patients with *P. falciparum* parasitaemia compared to those without parasitemia (*χ*^2^ = 3.93, *P* = 0.047) (Table 1). Diazepam also stopped seizures in less children with a primary diagnosis of malaria compared to those without malaria (40% vs. 62%, *χ*^2^ = 9.84, *P* = 0.002). However the level of parasitaemia did not influence the outcome and malaria was not an independent risk factor for the lack of termination of seizures (Table 2). Furthermore diazepam was not significantly associated with more episodes of respiratory depression in children with malaria compared to those without malaria (3.7% vs 1.1%, Fishers Exact Test = 0.189).

## Discussion

This study showed that diazepam is less efficacious in stopping seizures in children with falciaprum malaria. However, after adjusting for risk factors, the effect of malaria was not statistically significant (Table 2). In a study conducted in Uganda by Mpimbaza et al. (2008) the authors have shown that rectal diazepam is less effective in terminating convulsions in children with malaria. This result is in agreement with previous findings in Kenyan children which indicated that rectal absorption of parenteral diazepam is erratic and terminates less seizures than intravenous administration (Ogutu et al., 2002). Intramuscular paraldehyde does not act on benzodiazepine receptors; it is however used as an alternative first line drug to IV diazepam.

Patients with cerebral malaria have reduced plasma and CSF concentrations of histidine an amino acid which is an important constituent of the GABA-A receptor (Mturi et al., 2003), and this may influence benzodiazepine ligand binding (Wieland et al., 1992). This could be a plausible explanation to diazepam's decreased binding affinity at the GABA-A receptors (Kokwaro et al., 1997) or its reduced clinical efficacy in patients with parasitaemia.

The effect of diazepam on respiration also depends upon the integrity of the benzodiazepine receptors (Braestrup et al., 1982). The incidence of respiratory depression in this study is lower than that previously reported (Norris et al., 1999; Prensky et al., 1967) and may reflect the decreased receptor sensitivity in malaria patients. However there were insufficient numbers to determine if this occurred significantly less frequently in children with malaria.

The difference in diazepam efficacy is not only seen in the comparison between the malaria group versus the non-malaria group but those patients with and without parasitaemia as a whole. Thus diazepam may be less efficacious in controlling seizures in children with malaria. However a randomized trial is needed to clarify these findings and to elucidate the effect of malaria on benzodiazepine receptors in humans.

## Figures and Tables

**Table 1:** Treatment outcome of children given diazepam to stop acute seizures

	Number given diazepam, *N* = 200	Number (%) in whom seizures stopped
Patients without *P. falciparum* parasitaemia	53	32 (60%)
Patients with *P. falciparum* parasitaemia	147	69 (47%)
Patients with *P. falciparum* parasitaemia and clinical diagnosis of malaria	109	44 (40%)

**Table 2:** Multiple logistic regression analysis of factors associated with termination of seizures

	Seizures stopped, *N* = 298	Adjusted odds ratio	95% Confidence interval (CI)	*P*-value
Drug: (termination of seizures)		0.32	0.18–0.56	<0.001*
Diazepam vs.	103			
Paraldehyde	195			

Sex: female	139	1.53	0.90–2.61	0.129

History of seizures		1.10	0.57–2.10	0.767
Yes	199			
No	67			
Missing	32			

Length of seizure after drug administration				
<5 min^a^				
5–15 min	142	0.82	0.40–1.67	0.586
>15 min	39	0.13	0.06–0.29	<0.001*

Age				
0–6 months^a^				
6–12 months	44	0.57	0.20–1.65	0.308
1–3 years	52	0.54	0.18–1.62	0.275
3–6 years	136	0.64	0.24–1.69	0.370
6–13 years	26	2.41	0.51–11.3	0.262

Malaria: Yes	126	0.79	0.44–1.42	0.447

a This refers to the baseline group in the different categories.

* Represents a significant *P*-value.

## References

[bib1] Anon., 1993. Guidelines for epidemiologic studies on epilepsy. Commission on Epidemiology and Prognosis, International League Against Epilepsy. Epilepsia 34, 592–596. 10.1111/j.1528-1157.1993.tb00433.x 8330566

[bib2] Asindi A.A., Ekanem E.E., Ibia E.O., Nwangwa M.A. (1993). Upsurge of malaria-related convulsions in a paediatric emergency room in Nigeria. Consequence of emergence of chloroquine-resistant *Plasmodium falciparum*. Trop. Geogr. Med..

[bib3] Braestrup C., Schmiechen R., Neef G., Nielsen M., Petersen E.N. (1982). Interaction of convulsive ligands with benzodiazepine receptors. Science.

[bib4] Idro R., Gwer S., Kahindi M., Gatakaa H., Kazungu T., Ndiritu M., Maitland K., Neville B.G., Kager P.A., Newton C.R. (2008). The incidence, aetiology and outcome of acute seizures in children admitted to a rural Kenyan district hospital. BMC Pediatr..

[bib5] Kokwaro G., Edwards G., Roberts P., Ward S., Winstanley P., Watkins W. (1997). Infection with *Plasmodium berghei* alters benzodiazepine receptor in rat brain. Arch. Med. Res..

[bib6] Mbogo C.M., Mwangangi J.M., Nzovu J., Gu W., Yan G., Gunter J.T., Swalm C., Keating J., Regens J.L., Shililu J.I., Githure J.I., Beier J.C. (2003). Spatial and temporal heterogeneity of Anopheles mosquitoes and *Plasmodium falciparum* transmission along the Kenyan coast. Am. J. Trop. Med. Hyg..

[bib7] Molyneux M.E., Taylor T.E., Wirima J.J., Borgstein A. (1989). Clinical features and prognostic indicators in paediatric cerebral malaria: a study of 131 comatose Malawian children. Q. J. Med..

[bib8] Mpimbaza A., Ndeezi G., Staedke S., Rosenthal P.J., Byarugaba J. (2008). Comparison of buccal midazolam with rectal diazepam in the treatment of prolonged seizures in Ugandan children: a randomized clinical trial. Pediatrics.

[bib9] Mturi F.N., MacClennan C., Keir G., Newton C.R.J.C. (2003). The blood brain barrier is impaired in Kenyan children with severe falciparum malaria. J. Cereb. Blood Flow Metab..

[bib10] Mwangi T.W., Ross A., Snow R.W., Marsh K. (2005). Case definitions of clinical malaria under different transmission conditions in Kilifi District, Kenya. J. Infect. Dis..

[bib11] Norris E., Marzouk O., Nunn A., McIntyre J., Choonara I. (1999). Respiratory depression in children receiving diazepam for acute seizures: a prospective study. Dev. Med. Child Neurol..

[bib12] Ogutu B.R., Newton C.R., Crawley J., Muchohi S.N., Otieno G.O., Edwards G., Marsh K., Kokwaro G.O. (2002). Pharmacokinetics and anticonvulsant effects of diazepam in children with severe falciparum malaria and convulsions. Br. J. Clin. Pharmacol..

[bib13] Prensky A.L., Raff M.C., Moore M.J., Schwab R.S. (1967). Intravenous diazepam in the treatment of prolonged seizure activity. N. Engl. J. Med..

[bib14] Qureshi A., Wassmer E., Davies P., Berry K., Whitehouse W.P. (2002). Comparative audit of intravenous lorazepam and diazepam in the emergency treatment of convulsive status epilepticus in children. Seizure.

[bib15] Reyburn H., Mbatia R., Drakeley C., Bruce J., Carneiro I., Olomi R., Cox J., Nkya W.M., Lemnge M., Greenwood B.M., Riley E.M. (2005). Association of transmission intensity and age with clinical manifestations and case fatality of severe *Plasmodium falciparum* malaria. JAMA.

[bib16] Waruiru C.M., Newton C.R., Forster D., New L., Winstanley P., Mwangi I., Marsh V., Winstanley M., Snow R.W., Marsh K. (1996). Epileptic seizures and malaria in Kenyan children. Trans. R. Soc. Trop. Med. Hyg..

[bib17] Wieland H.A., Luddens H., Seeburg P.H. (1992). A single histidine in GABAA receptors is essential for benzodiazepine agonist binding. J. Biol. Chem..

